# The Computerized Adaptable Test Battery (BMT-*i*) for Rapid Assessment of Children's Academic Skills and Cognitive Functions: A Validation Study

**DOI:** 10.3389/fped.2021.656180

**Published:** 2021-07-08

**Authors:** Catherine Billard, Eric Thiébaut, Sahawanatou Gassama, Monique Touzin, Jean-Christophe Thalabard, Anne Mirassou, Arnold Munnich

**Affiliations:** ^1^Association pour la Recherche sur les Troubles des Apprentissages (ARTA), Paris, France; ^2^Laboratoire Lorrain de Psychologie et Neurosciences de la Dynamique des Comportements (2LPN), Université de Lorraine (EA 7489), Nancy, France; ^3^Centre Ressources sur les Troubles des Apprentissages Paris Santé Réussite, Paris, France; ^4^Université de Paris-Sorbonne Paris Cité, Imagine Institute, INSERM UMR1163, Paris, France

**Keywords:** test screening, learning disabilities, academic skills, cognitive functions, child

## Abstract

**Background:** Learning disabilities in children are a major public health concern worldwide, having a prevalence of 8%. They are associated with lost social, educational, and ultimately, professional opportunities for individuals. These disabilities are also very costly to governments and raise the issue of the appropriate means of screening. Unfortunately, validated tools for preliminary appraisal of learning and cognitive function in struggling children are presently restricted to specific age ranges and cognitive domains. This study sought to validate a first-line battery for assessment of academic skills and cognitive functions.

**Materials and Methods:** The computerized Adaptable Test Battery, or BMT-*i*, includes a panel of tests for the first-line assessment of children's academic skills and cognitive functions. The tests reflect expected abilities for the age group in question, exploring academic skills (written language and mathematical cognition) and cognitive domains (verbal, non-verbal, and attentional/executive functions). The authors relied on the results of these tests for a sample of 1,074 Francophone children representative of the mainland French school-age population (522 boys and 552 girls, ages 4–13, from 39 classes at 7 public and 5 private schools). Thirteen speech-language pathologists and neuropsychologists individually administered the tests.

**Results:** The psychometric characteristics of the empirical data obtained showed acceptable to good test homogeneity, internal consistency (Cronbach's alpha: > 0.70), test-retest reliability (intraclass correlation coefficients: ~0.80), and consistency with reference test batteries (*r*: 0.44–0.96).

**Conclusion:** The BMT-*i* was validated in a large sample of children in mainstream French schools, paving the way for its use in first-line screening of learning disabilities among children with complaints, whether their learning difficulties have been flagged by their parents or by their teachers.

## Introduction

Because of their high prevalence (8% among children 3–17 years old) (1), learning disabilities are a public health priority worldwide. They frequently concern several cognitive dimensions—written and oral language skills, mathematics, drawing and handwriting, motor function, visuospatial skills, and attentional as *well* as executive functions—justifying the need for a comprehensive view (2–4). The variety of terms associated with these conditions (e.g., *disorder, disability, difficulty*, and *slow learner*) illustrates the diversity of perspectives and makes it harder to share knowledge about them (5).

The emergence of cognitive sciences has enriched the theoretical models applied for the identification and evaluation of learning disabilities (LD). In the last 50 years, authors have developed integrative models considering (i) academic skills, (ii) underlying cognitive skills, and (iii) neurobiological correlates, including familial forms and environmental factors (6).

There is a growing consensus in support of early identification of LD by standardized tests and appropriate pedagogical interventions (7–10). Phased implementation of screening (6) is essential for the identification of learning disabilities and their effective remediation—such as through evidence-based pedagogical interventions, the long-term benefits of which have been extensively demonstrated (11–14). To meet the demands of clinical practice, screening tools must be language-specific and exhibit acceptable psychometric properties and sensitivity. Following their use, more focused assessments—conducted by speech therapists, psychomotor therapists, occupational therapists, or neuropsychologists, depending on the learning area affected—may be prescribed (9, 10, 14–16).

The computerized Adaptable Test Battery (BMT-*i*) is a panel of tests for the first-line assessment of children's academic skills and cognitive functions, from kindergarten (age 4) to seventh grade (age 13). Designed as an adaptable set of tests suitable for a comprehensive evaluation, the BMT-*i* succeeds the Battery for Rapid Evaluation of Cognitive Functions (Batterie Rapide d'Evaluation des Fonctions Cognitives, or BREV) originally designed to provide health professionals with a quick clinical tool for screening acquired and developmental cognitive deficits in children ages 4–8 (17, 18). Including tests in five domains that evaluate the various cognitive components concerned by LDs (4), the computerized BMT-*i* permits broader exploration of written language abilities (reading fluency, reading comprehension, and spelling), mathematical cognition (numbers, arithmetic, and problem-solving), and three cognitive domains (verbal, non-verbal, and attentional/executive functions). BMT-*i* tests assess the skills expected to be acquired by children in their respective age groups, between the ages of 4 and 13. They are meant to be simple to administer, short (10–30 min per domain, depending on age), and easy to score, and they can be taken at school or during an appointment with a health professional. Their purpose is rapid identification of children in the general population who require specialized assessments for precise diagnosis of LD, as recommended by France's Haute Autorité de Santé (HAS) (15). Standards defined by the American Educational Research Association (AERA), the American Psychological Association (APA), and the National Council on Measurement in Education (NCME) have guided test design and contributed to their validity (19).

Here we report psychometric data on the validity of the BMT-*i* using a sample of over a thousand French-speaking children—without prior complaints or previously identified LDs—representative of the mainland French school-age population.

## Population and Methods

### BMT-*i* Description

Design of the BMT-*i* has proceeded in several steps since 2010. Over the last 5 years, it has been gradually implemented, stratified by age groups and cognitive functions, and finally computerized. BMT-*i* tests apply neuropsychological models for a separate first-line examination of each of the five major domains of *academic skills*—i.e., (i) written language (reading fluency, reading comprehension, and spelling) (20) and (ii) mathematical cognition (numbers, arithmetic, and problem–solving) (21)—and *cognitive function*—i.e., (iii) oral language (vocabulary, grammar, and phonological skills) (22), (iv) non-verbal functions (reasoning, drawing, handwriting, and visuospatial construction), and (v) attentional/executive functions (see Table 1 and Supplementary Data). For this last domain, the computerization of BMT-*i* tests allows objective standardized measures of the scores in the main attentional/executive processes (sustained and selective attention, flexibility and inhibition, working memory). While the academic aptitude tests are adapted to each grade level, most of the cognitive function tests are identical across a given group, i.e., “youngest” (kindergarten through first grade), “intermediate” (second through fourth grade), or “oldest” (fifth through seventh grade). Scores are instantly and automatically converted into normed results that are summarized in a report. The BMT-*i* is intended for use by trained health professionals and their teams, including pediatricians, child psychiatrists, school doctors, general practitioners, psychologists, specialized professionals such as speech therapists, psychomotor therapists and occupational therapists. The published versions of the BMT-*i* tests (23) are described in the Supplementary Data.

**Table 1:** Overview of BMT-*i* tasks.

**Skills**	**Kindergarten**	**Grade 1**	**Grades 2–4**	**Grades 5–7**
Written language: Reading	Letters	Trimesters 1–3: decoding Trimester 3: reading time, errors, comprehension	One of two texts: reading time, errors, and comprehension	One of two texts: reading time, errors, and comprehension
Written language: Dictation	Letters	Letters, syllables, one sentence	Pseudowords, sentences	Text
Mathematical cognition	Numbers: quantification, reading, dictation; Problems	Numbers: reading, dictation, analog representation; Calculation; Problems	Numbers: reading, dictation, analog representation; Mental calculation/math fluency; Problems	Numbers: reading, dictation, analog representation; Base-10 representation; Mental calculation/math fluency; Problems
Oral language	Phonological awareness, non-word repetition, lexical production and comprehension, syntactic expression and comprehension		Non-word repetition, lexical production and comprehension, syntactic expression and comprehension	Non-word repetition, lexical production and comprehension, syntactic expression, and comprehension
Non-verbal: Reasoning	Pattern completion test (20 matrices)		Pattern completion test (24 matrices)	Pattern completion test (24 matrices)
	Classification test			
Non-verbal: Drawing, handwriting, visuospatial construction	Copying 6 simple figures	Copying 5 simple figures	Copying 5 simple figures	
			Copying a complex figure Dictation handwriting score	
	Block construction task		Block construction task	
Attention/executive functions			Sustained visual attention Controlled auditory attention	
		Digit span (forward and backward)		

### Population Recruitment

**Figure 1:**
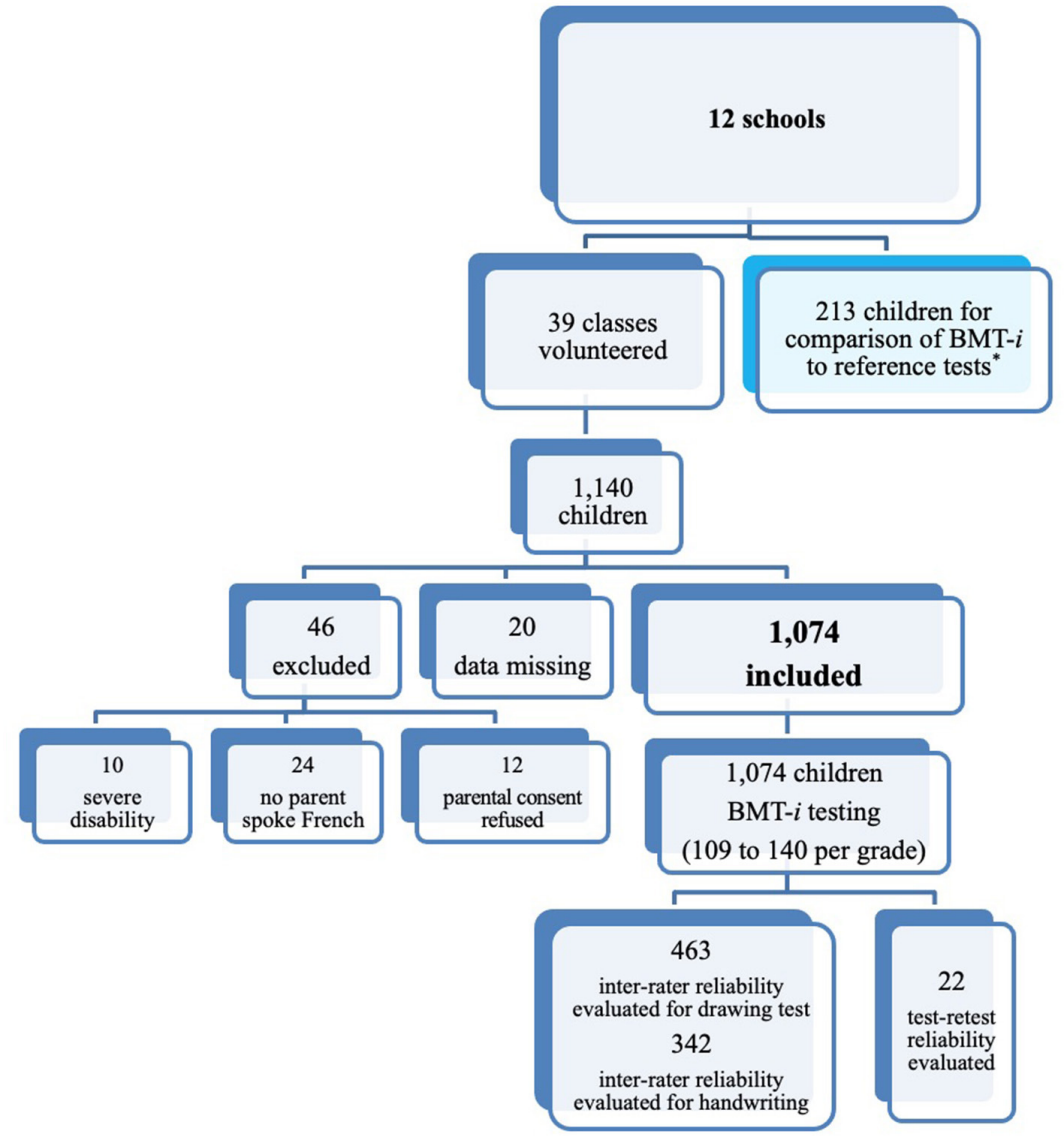
Recruitment of participants. *For comparison with BMT-*i* tests, reference tests were administered to 213 children: 44 third graders and 96 middle schoolers for written language assessment, and 73 children grades 1–7 for pattern completion.

The rational for the sample size for BMT-*i* corresponded to a classical approach in a descriptive study for obtaining an estimation of a prevalence p with both a specified precision (0.05) and a chosen degree of confidence (0.95). The children were exposed to an adapted testing corresponding to their grade, categorized into three levels depending on their age (kindergarten, elementary-school, middle school). Figure 1 describes the target population.

This prospective study included 1,074 children aged 4–13 (522 boys and 552 girls) from 12 mainstream public or private schools across France (Greater Paris, Toulouse, Orleans, and rural areas). The 12 schools voluntarily participated and represented the diversity of their geographic (urban, suburban, or rural) and socioeconomic environments. After approval was granted by their respective regional education authorities and 99% of parents gave informed consent, teachers agreed that children in their classrooms would be tested in alphabetical order. All children were tested except those (i) severely handicapped, (ii) having no parent who spoke French, or (iii) whose parents did not consent to the tests (see Figure 1: 5.8% of the initial sample).

### Methods

#### BMT-*i* Testing

Tests were administered during the 2015–2016 academic year. During each of the three trimesters of the French academic year, a third of the participating children were tested—with the exception of the younger kindergartners (middle kindergarten section, ages 4 and 5), who began testing in February.

The tests were administered in a single session (average duration: 45 min) for kindergartners; two sessions for elementary-school students (average total duration: 90 min); and because of the greater number of mathematical cognition tests for their age group, three sessions for middle-school students (average total duration: 120 min).

The job category of each parent was recorded, using the nomenclature of the French National Institute of Statistics and Economic Studies (INSEE) (24). The most socioeconomically privileged job category for each household was used for grouping into three categories: “underprivileged” (manual workers, non-managerial employees, unemployed), “average” (higher–level non-managerial professionals, farmers, artisans, storekeepers, and small business owners), and “privileged” (managers, executives, engineers, and other knowledge workers). Households were considered bilingual if they met the INSEE criterion, i.e., one of the two parents spoke a language other than French.

Tests were individually administered by an examiner from a group of eight speech-language pathologists and five neuropsychologists, who had received two sessions of collective training. The testing took place in a designated room of each school on a Microsoft Surface Pro 3 convertible laptop running Windows 8. Instructions for each test were displayed on the screen, and the examiner also provided explanations to children, especially the youngest. For the sake of consistency, items that had to be read to the children were recorded in advance, and the recordings were played back by the application. The only exceptions were dictation and reading questions, for which the child's pace had to be considered. Because the tests were computerized, response times could be recorded by the computer. This is particularly important in the assessment of attention and executive functions, where response times are measured to the nearest mils. Children's responses were recorded automatically, when touchscreen input was possible, or manually by the examiner, for oral responses or when more complicated, explicit scoring was required. Scores were instantly and automatically converted into normed results.

Examiners participated in semimonthly review meetings led by the authors, and frequently asked questions were regularly published to address potential scoring ambiguities. A clinical research assistant verified inclusion conditions (stratification), observance of the protocol, and thoroughness of tests. After anonymized data were exported, three of the neuropsychologist examiners performed double scoring of study logs under the authors' supervision.

#### Inter-Rater and Test-Retest Reliability

The scoring of most tests was objective and unbiased as responses were either automatically recorded or had clear answers (written language, mathematical cognition, reasoning and attention tasks). For scoring of participants' reproductions of simple or complex figures (463 children) and of handwriting (342 children), grade-specific inter-rater reliability coefficients were calculated using a random sample (Figure 1).

The 10th child on each class list of students was scheduled to be retested for the entire battery and by the same examiner 3 weeks later under strictly identical conditions. At the request of the teachers, the planned retest could only be conducted among kindergarten and elementary school children assessed in the third quarter of the school year in three schools. Therefore, the retested subsample consisted of 22 children (10 boys and 12 girls) aged 4.8–11.3 years and belonging to one of the three groups of classes: (i) kindergarten through first grade, (ii) second through fourth grades, and (iii) fifth grade (Figure 1).

#### Comparison With Other Tests

An additional study was conducted within the same schools to compare the consistency of the BMT-*i* with standardized reference test batteries commonly used in clinical practice (Figure 1). Children were arbitrarily selected to take reference tests that assessed the same functions, according to age-specific standards, within 2 weeks of taking the BMT-*i*. To compare written language tests, the authors administered the standardized tests used by French speech therapists—for reading, *Quelle Rencontre* (25) and *Le Vol du PC* (26); and for dictation, *Chronosdictées* (27) and *Le Corbeau* from the L2MA test battery (28)—to 44 third graders (26 boys and 18 girls, 8.1–9.2 years old) and 96 middle schoolers (50 boys and 46 girls, 10.8–13.1 years old). For pattern completion, the BMT-*i* was compared to WISC-V Fluid Reasoning subtests, including Matrix Reasoning, administered to 73 children (48 boys and 25 girls, 6.5–13.5 years old, grades 1–7) (29).

#### Statistical Analyses

The inter-rater reliability coefficients for drawing and handwriting assessment were calculated and evaluated using correlation and linear regression coefficients.

Test-retest reliability was measured using the intraclass correlation coefficient, which considers school level to be a fixed covariate measure (30). An intraclass correlation coefficient between 0.50 and 0.75 indicates an average level of reliability; > 0.75 and ≤ 0.90, a good level; and >0.90, an excellent level (30).

Test item homogeneity was analyzed using DIMTEST (31) for dichotomous variables and LISREL (32) uni-dimensionality tests for the others. Score reliability was measured by Cronbach's alpha (33), where ≥ 0.70 indicates a good level of reliability (34).

In addition, the quality of fit between the theoretical model and the empirical data was estimated through confirmatory factor analysis using the Root Mean Square of Error Approximation (RMSEA). RMSEA values of <0.08 are deemed acceptable (35). Analyses were conducted by grade level because of the use of age-specific items for the different domains.

Statistical analysis of the test battery comparison included correlation of raw scores (correlation and linear regression coefficients). Degree of agreement was determined by calculating Cohen's kappa: values in the range of 0.21–0.40 indicate fair agreement; 0.41–0.60, moderate; 0.61–0.80, substantial; and 0.81–1.00, almost perfect (36). For the purpose of comparison, scores on the BMT*-i* and reference tests scores were categorized as very low (7th percentile or lower), low (7th through 20th percentile), or normal (>20th percentile).

Analyses were carried out using JMP software (37) and the lme4 statistical package for R (38).

## Results

### Sample Demographics

Table 2 summarizes demographic data demonstrating the representativeness of the sample. The job category distribution, specified for 95% of the sample, differed between student age groups: the proportion of “privileged” households diminished (except for fourth grade [CM1]) from kindergarten to middle school (*p* < 0.001). The overall proportion of underprivileged families (29%) was similar to that reported in a recent French perinatal survey (28%) (39). It was very unlike the job category distribution published by the INSEE (24), which may be explained by the different age profile of parents in the present study. For 73% of the children in the sample, both parents spoke only French: this is similar to the INSEE's finding (40). In 6% of the cases, children had undergone reeducation or therapy before the test, and in 2% of the cases, children were still receiving such support at the time of testing. Very few students had repeated (0.6%) or skipped (1.4%) a grade.

**Table 2:** Characteristics of normative sample.

**Grade**	**Number of children**	**Mean age (range), years**	**Boys (%)**	**Girls (%)**	**JC^**a**^ (%)**	**Bilingualism^**b**^**	**Support^**c**^**
KG2	112	4.8 (4.1–5.4)	52 (46%)	60 (54%)	1 (12%) 2 (15%) 3 (70%)	16%	1%
KG3	134	5.6 (4.8–6.4)	66 (49%)	68 (51%)	1 (14%) 2 (19%) 3 (66%)	22%	2%
1	124	6.6 (5.8–7.4)	53 (43%)	71 (57%)	1 (23%) 2 (15%) 3 (58%)	30%	3%
2	109	7.6 (6.6–8.4)	53 (49%)	56 (51%)	1 (25%) 2 (26%) 3 (47%)	26%	9%
3	111	8.7 (7.9–9.3)	58 (52%)	53 (48%)	1 (44%) 2 (15%) 3 (34%)	31%	8%
4	110	9.6 (8.4–10.6)	48 (44%)	62 (56%)	1 (22%) 2 (32%) 3 (43%)	23%	9%
5	105	10.7 (9.8–11.3)	47 (45%)	58 (55%)	1 (33%) 2 (29%) 3 (34%)	27%	13%
6	129	11.5 (10–12.3)	66 (51%)	63 (49%)	1 (40%) 2 (28%) 3 (23%)	35%	15%
7	140	12.4 (11.2–13.7)	79 (56%)	61 (44%)	1 (45%) 2 (28%) 3 (15%)	35%	15%
Total	1,074	8.7 (4.1–13.7)	522 (49%)	552 (51%)	1 (29%) 2 (23%) 3 (43%)	27.2%	8%

a *JC = job category, specified for 95% of the sample. 1 = under privileged, 2 = average; 3 = privileged*.

b *Bilingualism (%), i.e., one of two parents speaks language other than French*.

c *Undergoing reeducation or receiving remedial support. KG2 = 2nd year of kindergarten, KG3 = 3rd year of kindergarten*.

### Reproducibility of Scores

#### Inter-Rater Reliability

Inter-rater reliability coefficients for a random sample revealed stable scores on the figure copying (*r*: 0.77–0.97) and handwriting (*r*: 0.76–0.84) assessments. Correlations and regression coefficients were significant for all grades (Table 3).

**Table 3:** Inter-rater reliability coefficients for drawing and handwriting scores.

**Skill**	**Grade (*n*)**	**Score 1 mean (sd)**	**Score 2 mean (SD)**	*R*^2*^	r^*^
**Copying simple figure** (scored over 5 or 6 according to grade)	KG (131)	3.87(1.04)	3.97(1.1)	0.69	0.79
	1 (19)	1.6(0.96)	2.1(1.1)	0.80	0.91
	2 (108)	2.94(0.92)	2.93(0.92)	0.82	0.91
	3 (37)	2.02(1.2)	1.95(1.14)	0.86	0.93
	4 (34)	2.92(1.06)	2.68(1.06)	0.77	0.87
**Copying complex figure** (scored over 13)	2 (108)	4.69(2.2)	4.62(2.1)	0.78	0.88
	3 (37)	4.75(1.62)	4.95(1.62)	0.89	0.95
	4 (34)	6.1(1.79)	6.3(2.35)	0.59	0.77
	5 (32)	5.6(2.5)	5.4(2.48)	0.94	0.97
	6+7 (102)	4.9(2.7)	5.1(2.2)	0.94	0.97
**Handwriting** (scored over 6)	2 (63)	1.40(1.4)	1.42(1.3)	0.61	0.84
	3 (64)	1.7(1.7)	2.1(1.8)	0.62	0.77
	4 (62)	1.1(1.3)	1.2(1.35)	0.53	0.76
	5 (61)	1.13(1.3)	1.0(1.1)	0.45	0.78
	6+7 (92)	1.01(1.2)	1.4(1.4)	0.56	0.76

* *All R^2^ and r values were significant (p < 0.0001). KG, kindergarten; SD, standard deviation*.

#### Test-Retest Reliability

Table 4 shows the intraclass correlation coefficients for each test. Most coefficients ranged from 0.8 to 0.9, corresponding to a good level of reliability. None were below 0.67. Differences between values for the 2.5th and 97.5th percentiles were relatively small.

**Table 4:** BMT-*i* test–retest reliability measured by intraclass correlation coefficients.

**Skills**		Number of students retested^*^	**ICC, 2.5th percentile**	**ICC, median**	**ICC, 97.5th percentile**
Written language	Reading time	14	0.908	0.971	0.991
	Reading errors	14	0.678	0.888	0.963
	Reading comprehension	14	0.720	0.887	0.961
	Dictation errors	14	0.673	0.882	0.956
	Dictation time	14	0.687	0.886	0.960
Mathematical cognition	Number dictation	15	0.684	0.888	0.960
	Number reading	21	0.702	0.886	0.960
	Addition fluency	14	0.699	0.883	0.959
	Subtraction fluency	14	0.693	0.886	0.960
	Multiplication fluency	11	0.691	0.888	0.962
	Problem-solving	14	0.689	0.892	0.961
	Composite score	21	0.684	0.885	0.963
Oral language	Lexical deployment	21	0.703	0.889	0.961
	Lexical comprehension	22	0.691	0.888	0.961
	Syntactic completion	18	0.681	0.886	0.962
	Sentence repetition (words)	14	0.694	0.889	0.961
	Sentence repetition (morphemes)	14	0.698	0.887	0.960
	Syntactic comprehension	22	0.697	0.888	0.960
	Phonology	20	0.711	0.889	0.959
Nonverbal functions	Complex figure score	14	0.689	0.884	0.958
	Complex figure time	14	0.691	0.890	0.963
	Handwriting	14	0.710	0.887	0.960
	Pattern completion score	21	0.683	0.888	0.959
	Pattern completion time	21	0.680	0.889	0.963
Visual attention	% negative errors	15	0.700	0.889	0.959
	% positive errors	15	0.679	0.883	0.962
	Reaction time (median)	15	0.692	0.889	0.960
	Reaction time (SD)	15	0.699	0.888	0.959
Auditory attention	Part A—CA	14	0.691	0.885	0.961
	Part B, flexibility—CA	14	0.684	0.886	0.959
	Part B, triangle—CA	14	0.691	0.887	0.960

* *Number of students tested varied from 11 to 22 between age groups. ICC, intraclass correlation coefficient*.

#### Uni-Dimensionality and Internal Consistency

The authors first sought to evaluate the hypothesis of test uni-dimensionality for the 1,074 participating children—that is, to confirm that each of the relevant tests did indeed evaluate the same aspect of the skill in question. For most if not all grades, tests of mathematical cognition, auditory attention, oral language, and non-verbal function (except for the figure copying test taken by the oldest kindergartners, which included the three most complicated figures) were uni-dimensional. For children in kindergarten and elementary school, due to the limited number of mathematical test items, composite scores were assigned.

Table 5 shows values of Cronbach's alpha, reflecting the degree of internal consistency for BMT-*i* scores, and Table 6 gives means and standard deviations for tests whose format did not permit calculation of Cronbach's alpha. In the area of written language, reliability of scores for decoding among older kindergartners and first graders, and of total scores for dictations, was good to excellent. In the area of mathematical cognition, for all classes, composite scores based on the results of the main subtests demonstrated a good level of reliability. The same is true of accuracy scores obtained for mental math operations and comparison of number representations, and in middle school, for the various subtests. Scores on most of the verbal tests, the two reasoning tests, and the auditory attention test also indicated a good level of reliability. On block construction tests, levels of reliability were excellent in all classes for time to completion, and good (older kindergartners and first graders) or satisfactory (second to fifth graders) for accuracy. With regards to drawing tests, the level of reliability was good for time to completion, but insufficient for accuracy scores.

**Table 5:** Internal consistency of BMT-*i* test scores.

**Skill**			**Grade**							
			**KG**	**1**	**2**	**3**	**4**	**5**	**6 & 7**
**WRITTEN LANGUAGE**									
Decoding			0.84	0.94					
Dictation			0.67	0.82	0.78	0.70	0.86	0.89	
**MATHEMATICAL COGNITION**									
Number dictation									0.79
Base 10									0.83
CNR, % CA				0.73	0.86				
Mental math		Addition			0.89				
		Subtraction			0.92				
		Multiplication				0.88			
		Division					0.89		
		Mixed					0.82		
Composite score			0.71	0.79	0.71	0.75	0.70	0.70	0.88
**ORAL LANGUAGE**									
Lexical deployment, CA			0.87		0.85			0.85	
Lexical comprehension		CA	0.79		0.73			0.71	
		Time (s)	0.75		0.86			0.89	
Sentence repetition		Words, CA			0.73			0.77	
		Morph., CA			0.67			0.74	
Sentence completion			0.73		0.72				
Syntactic comprehension			0.73		0.70			0.62	
**NON-VERBAL FUNCTIONS**									
Simple figures copy		Quality	0.56	0.61		0.45			
		Time	0.61	0.76		0.82			
Complex figure copy		Quality			0.51				
Block construction		CA	0.85		0.67				
		Time	0.86		0.88				
Pattern completion		CA	0.73		0.77			0.74	
		Time	0.82		0.82			0.87	
Classification			0.94						
**ATTENTION AND EXECUTIVE FUNCTIONS**									
Auditory attention	A	CA			0.77				
	B	Flexibility			0.73				
		Triangle			0.74				
		Inhibition			0.92				
Span				0.62					

*Numbers in table are values of Cronbach's alpha. CA, correct answers; CNR, comparison of number representations; KG, kindergarten; morph, morphemes*.

**Table 6:** Reliability of tests for which Cronbach's alpha not calculable.

**Skill**		**Grade**						
		**KG**	**1**	**2**	**3**	**4**	**5**	**6 & 7**
**WRITTEN LANGUAGE**									
Text reading	Time (s)			83 (36)	135 (45)	167 (40)	244 (53)	235 (51)
	Errors (*n*)			2.8 (4)	3.6 (3)	4.1 (4.7)	7 (7)	7 (6)
	Comprehension			7 (2.7)	11.6 (3)	19 (4.4)	12.8 (4)	12.7 (3.8)
**ORAL LANGUAGE**									
Phonology		15 (2)	16 (1)	16 (1)	16.6 (1)	16.6 (1)	18.1 (1.4)	
**NONVERBAL FUNCTIONS**									
Complex figure	Time (s)			109 (42)	110 (31)	106 (35)	109 (35)	92 (40)
**ATTENTION AND EXECUTIVE FUNCTIONS**									
Visual attention	% NE			9 (8)	7.2 (6)	4.6 (4.2)	4.9 (4.9)	4.1 (4.6)
	% PE			38 (20)	41 (19)	37 (21)	41 (21)	43 (20)
	RT, med. (ms)			764 (132)	707 (118)	648 (142)	619 (141)	586 (93)
	RT, SD (ms)			445 (179)	444 (202)	355 (191)	342 (214)	396 (224)

*Values given as mean (standard deviation). KG, kindergarten; med, median; NE, negative errors (i.e., no answers given); PE, positive errors (i.e., wrong answers given); RT, reaction time; SD, standard deviation*.

### Consistency of Empirical Data With Theoretical Model

Table 7 presents RMSEA values (0.036–0.075) indicating compatibility of scores for all tests—in the five areas of verbal, non-verbal, and attentional/executive functions; written language; and mathematics—and grades with the underlying theoretic model.

**Table 7:** Model validity coefficients for each grade.

**Grade**	**Number of children**	**χ^**2**^ (*p*)**	**df**	**χ^**2**^/df**	**RMSEA**	**(90% CI)**	**SRMR**
KG2	112	116 (0.002)	74	1.57	0.07	(0.045–0.096)	0.075
KG3	134	227 (0.00)	146	1.55	0.065	(0.048–0.081)	0.08
1	124	116 (0.002)	74	1.57	0.07	(0.045–0.096)	0.075
2	109	392 (0.03)	344	1.14	0.036	(0.008–0.052)	0.08
3	111	529 (<0.001)	370	1.43	0.06	(0.05–0.07)	0.08
4	110	691 (0.002)	428	1.61	0.075	(0.065–0.085)	0.11
5	105	435 (0.000)	294	1.48	0.068	(0.054–0.080)	0.12
6 & 7	270	1,252 (<0.001)	724	1.73	0.07	(0.07–0.08)	0.08

*χ^2^, chi–square; CI, confidence interval; df, degrees of freedom; KG2, 2nd year of kindergarten; KG3, 3rd year of kindergarten; SRMR, standardized root mean square residual (good if < 0.05; acceptable if < 0.08); RMSEA, root mean square of error approximation (good If <0.06; average if <0.08)*.

### Comparison of BMT-*i* With Reference Test Batteries

Table 8 shows that BMT-*i* scores for reading time, reading accuracy, and dictations were significantly correlated with reference test battery scores at both the middle-school and third-grade levels (*r* ≥ 0.78). For reading comprehension, the correlation between BMT-*i* and reference tests scores was high at the third-grade level (*r* = 0.78) and average for the two BMT-*i*'s texts at middle-school (text 1: *r* = 0.47 and text 2 *r* = 0.57). There is an average correlation between BMT-*i* pattern completion test scores and the WISC-V Matrix Reasoning subtest (*r* = 0.57) and Fluid Reasoning Index (*r* = 0.44), respectively. Table 8 also indicates agreement (Cohen's kappa) between the classifications of BMT-*i* and reference test battery scores into three groups (very low, low, and normal). Cohen's kappa values were moderate (0.39–0.68) for all tests except middle-school reading comprehension (0.25 and 0.30) and pattern completion (0.24), for which they were acceptable. All Cohen's kappa values were significant, with *p*-values ranging from < 0.0001 to < 0.01.

**Table 8:** Comparison of BMT-*i* and reference tests.

**Test**		***n***	**Mean**	**SD**	**min–max**	***R*^**2**^**	***r***	**Cohen's kappa (CI)**
**Reading**^**a**^**–middle school**									
BMT-*i* text 1	Time (s)	49	233.5	56	156–473	0.93^***^	0.96^***^	0.58^***^ (0.3–0.8)
BMT-*i* text 2		47	238.7	68.7	168–565	0.74^***^	0.86^***^	0.53^***^ (0.24–0.8)
Ref		96	192.1	43.9	120–402			
BMT-*i* text 1	WCR/min	49	144	28.6	67–206	0.93^***^	0.96^***^	0.46^**^ (0.2–0.75)
BMT-*i* text 2		47	138	29.2	52–188	0.83^***^	0.91^***^	0.45^***^ (0.2–0.75)
Ref		96	131	2.36	58–201			
BMT-*i* text 1	Comp—score over 20	49	12.8	3.4	4.5–18.5	0.22^**^	0.47^***^	0.25^*^ (0.1–0.52)
BMT-*i* text 2		47	12.6	3.2	5.5–18.5	0.32^***^	0.57^***^	0.30^*^ (0.1–0.63)
Ref	Comp—score over 66	96	38.1	8.5	18–55.5			
**Dictation**^**b**^**–middle school**									
BMT-*i*	Errors	96	15.1	8.9	1.1–36			
Ref dictation 1	Errors	96	26.5	16.5	2.1–36	0.76^***^	0.87^***^	0.55^***^ (0.4–0.7)
Ref dictation 2	Score over 100	51	80.1	10.9	48–95	0.81^***^	0.9^***^	0.68^***^ (0.4–0.9)
**Reading**^**a**^**–grade 3**									
BMT-*i*	Time (s)	44	214	75	110–425			
Ref			230.4	74	123–449	0.88^***^	0.94^***^	0.39^**^ (0.25–0.8)
BMT-*i*	WCR/min		241	65	84–340			
Ref			274	45	216–342	0.87^***^	0.93^***^	0.41^**^ (0.20–0.6)
BMT-*i*	Comp		12.2	5.3	3.1–20			
Ref	Comp		8.8	2.7	3.1–14	0.61^***^	0.78^***^	0.42^**^ (0.2–0.63)
**Dictation**^**b**^**–grade 3**									
BMT-*i*	Errors	44	16.9	6.6	3.1–32			
Ref	Errors		32.9	13.9	2.2–63	0.71^***^	0.84^***^	0.39^**^ (0.13–0.6)
**Reasoning—grades 1–7**									
BMT-*i* PC	Score from 0 to 7	73	3	1.5	1–7			
WISC	Matrix NS	73	10	3	3–18	0.33^***^	0.57^***^	0.24^**^ (0.1–0.39)
WISC	FRI	73	100	19	67–144	0.2^***^	0.44^***^	

*Values of R^2^ and r for comparison of BMT-i and corresponding reference tests. BMT-i texts (grades 6 and 7): text 1 Star du Rap; text 2: Apprentie Sorcière*.

a *Reference texts: Quelle Rencontre (3rd grade) and Vol du PC (middle school)*.

b *Reference dictation 1: Chronosdictées; reference dictation 2: Le Corbeau*.

* *p < 0.01*;

** *p < 0.001*;

*** *p < 0.0001*;

*CI, confidence interval; comp, comprehension; FRI, Fluid Reasoning Index; NS, standard score; PC, pattern completion; ref, reference; SD, standard deviation; WCR/min, words correctly read per minute; WISC, WISC-V Matrix Reasoning*.

## Discussion

Here we report on the validity of psychometric data collected from a large sample of French children, without prior complaints or previously identified LDs, using a novel computerized battery of tests, the BMT-*i*. This single screening tool includes diverse tasks aimed at identifying the different aspects of LDs, as internationally recommended (4, 10, 14–16). Each test can be used separately with specific norms, allowing relevant tests to screen for one or more areas of complaint. Its computerized format has the merit of limiting measurement bias in the reporting and rating of children' responses for most subtests. In particular, the two attentional tests of the BMT-*i* are computerized and the global results are directly provided by an algorithm.

Inter-rater reliability coefficients, calculated to estimate the effect of subjectivity on the assessment of drawing and handwriting, confirm the stability of the total score (41). Despite the limited number of retests, intra-class correlation coefficients were appropriate for all tests—including those for which internal consistency was insufficient (30).

The uni-dimensionality of most of the tests (i.e., proof that each indeed evaluated the same aspect of the given aptitude) allows for dependable interpretation of scores as indicators of children's aptitudes for reading, spelling, math, and various cognitive functions (verbal, non-verbal, and attentional). The coefficients of internal consistency, describing test score reliability, are generally satisfactory, but scores on some tests, including for quality of drawing, were very unstable. Time to completion offers additional information about a child's skills, as long as it is carefully considered in the light of the quality score.

To verify the consistency of score data with the theoretical model and determine whether the five cognitive domains were accurately represented, confirmatory factor analyses were performed. These indicated that test scores were significantly related to the cognitive skills they theoretically represented. Hence, the results reported are aligned with the generally recognized theoretical structures associated with the five domains of academic skills and cognitive function (2, 4, 6, 10). It is worth noting certain relationships between test types. Reading comprehension scores form a group with oral language test scores but not with reading times or reading errors. At the middle-school level (sixth and seventh grades), all scores on written language tests are grouped with those for oral language tests. This grouping of reading comprehension with oral language skills is consistent with the different profiles of written language disorders described in the literature (dyslexia vs. poor reading comprehension) and with the links between oral language and reading comprehension skills (20, 42), and it justifies the need to assess both reading fluency and comprehension as well as oral language (43).

Comparison of BMT-*i* and reference tests revealed high levels of correlation in all areas of written language, except reading comprehension among middle schoolers, for which *r* values indicated average correlation. The correlation between the BMT-*i* pattern completion test scores and the WISC-V fluid reasoning subtests suggests the reliability of potential referrals for the indication of a psychometric assessment for which it is not a substitute. No comparisons were made in areas other than written language and reasoning.

Interpretation of these results must be tempered by recognition of the various limitations of the study. To begin with, the results of the reading comprehension assessment vary according to the nature of the tasks proposed, which points to a need for more precise tests. In addition, the reference tests selected were those available at the time of our study. Recent tests would have allowed a single, more elaborate battery to be used for all measures from second grade up (20, 44). The inter-rater reliability could not be determined for all subtests across the entire population owing to the diversity of population of schools where testers examined children. Furthermore, test-retest reliability could only be assessed for a group of 22 children.

The present validation of the BMT-*i* with a large sample of children representative of the diverse mainstream school population in France sets the stage for its use in first-line screening to identify LDs in children with difficulties flagged by parents or teachers. However, use in the diagnosis of LDs will require verification of its sensitivity, specificity, and predictive value, relative to other tests, in children with complaints. The BMT-*i* might be administered for preliminary cognitive assessment of children who are struggling in school, to properly refer them for specialized assessments.

The methods and tools employed for identification of LDs differ between countries and professions, and an international consensus has yet to be reached (5). LD screening tests are expected to be short and easy for non-specialized professionals to administer and interpret. Many tools that employ a language specific to the country in question and that target a particular domain are available to help identify children requiring a pedagogical intervention or specialized evaluation. The BMT-*i* is the only tool in French that meets this objective for all domains concerned, over a wide age range. For oral language, the reliability of current instruments is deemed insufficient to permit screening in young children without complaints (22); the quality of these instruments must be improved (45). Present methods for identifying reading difficulties are also imperfect (46, 47), ranging from a simple, carefully validated teacher questionnaire to the classic Wechsler Individual Achievement Test. Recent mathematics research insists on the importance of analyzing the different number manipulation and arithmetic skills (21, 48). Future development of computerized tests is expected (49). Moreover, the frequent comorbidities of LDs—namely handwriting, visuospatial (50), or attentional, and executive disorders (51, 52) deserve particular attention. In conclusion, the BMT-*i* can offer an initial appraisal of cognitive functions and help guiding children to specialized assessments and appropriate interventions (10). Hence, this study paves the road toward ongoing studies in populations with complaints. Getting help for LDs, which are inconsistently recognized, is an often expensive and complicated process, and the support that is received varies, but the BMT-*i* could make it more accessible and affordable.

## Data Availability Statement

The anonymized results and data of our research are available upon request to the first and corresponding author.

## Ethics Statement

Ethical review and approval was not required for the study on human participants in accordance with the local legislation and institutional requirements. Written informed consent to participate in this study was provided by the participants' legal guardian/next of kin.

## Author Contributions

CB and SG led the study and collected data. CB and J-CT (test-rest reliability) performed analyses. ET reviewed the analyses. AMi, J-CT, and AMu discussed the results. CB wrote the manuscript. MT and AMu revised the manuscript. All authors contributed to the article and approved the submitted version.

## Conflict of Interest

The authors declare that the research was conducted in the absence of any commercial or financial relationships that could be construed as a potential conflict of interest.

## References

[1] Cortiella C Horowitz SH. The State of Learning Disabilities: Facts, Trends, and Emerging Issues. 3rd ed. New York, NY: National Center for Learning Disabilities (2014).

[2] American Psychiatric Association. Diagnostic and Statistical Manual of Mental Disorders. 5th ed (DSM−5). Arlington, VA: American Psychiatric Publishing (2013). 10.1176/appi.books.9780890425596

[3] World Health Organization. The ICD−10 Classification of Mental and Behavioural Disorders: Clinical Descriptions and Diagnostic Guidelines. Geneva: WHO (1992). Available online at: https://apps.who.int/iris/handle/10665/37958

[4] Learning Disabilities Association of America. Types of Learning Disabilities. (2017). Available online at: https://ldaamerica.org/types-of-learning-disabilities/ (accessed on May 30, 2021).

[5] Grünke M Cavendish WM. Learning disabilities around the globe: Making sense of the heterogeneity of the different viewpoints. Learn Disabil Contemp J. (2016) 14:1–8. Available online at: http://docplayer.net/20930497-Learning-disabilities-around-the-globe-making-sense-of-the-heterogeneity-of-the-different-viewpoints.html

[6] Grigorenko EL Compton DL Fuchs LS Wagner RK Willcutt EG Fletcher JM. Understanding, educating, and supporting children with specific learning disabilities: 50 years of science and practice. Am Psychol. (2020) 75:37–51. 10.1037/amp0000452 31081650 PMC6851403

[7] Hale JB Kaufman A Naglieri JA Kavale KA. Implementation of IDEA: integrating response to intervention and cognitive assessment methods. Psychol Schools. (2006) 43:753–70. 10.1002/pim20186

[8] Fletcher JM Grigorenko EL. Neuropsychology of learning disabilities: the past and the future. J Int Neuropsychol Soc. (2017) 23:930–40. 10.1017/S1355617717001084 29198282 PMC6249682

[9] Hale JB Alfonso V Berninger V Bracken B Christo C Clark E. Critical issues in response-to-intervention, comprehensive evaluation, and specific learning disabilities identification and intervention: an expert white paper consensus. Learning Disability Q. (2010) 33:223–36. 10.1177/073194871003300310

[10] Schneider WJ Kaufman AS. Let's not do away with comprehensive cognitive assessments just yet. Arch Clin Neuropsychol. (2017) 32:8–20. 10.1093/arclin/acw104 27993770

[11] Torgesen JK. The prevention of reading difficulties. J School Psychol. (2002) 40:7–26. 10.1016/S0022-4405(01)00092-9

[12] Fuchs LS Vaughn S. Responsiveness-to-intervention: a decade later. J Learn Disabil. (2012) 45:195–203. 10.1177/0022219412442150 22539056 PMC3357628

[13] Reynolds AJ Sou–Ruu Ou Temple JA. A multicomponent, preschoool to third grade preventing intervention and educational attainment at 35 years of age. JAMA Pediatrics. (2018) 172:246–256. 10.1001/jamapediatrics.2017.4673 PMC5885840 29379955

[14] INSERM Collective Expertise Centre. INSERM Collective Expert Reports. Paris: Institut national de la santé et de la recherche médical. Dyslexia, Dysorthography, Dyscalculia: Review of the scientific data (2007). 21348173

[15] Haute Autorité de Santé (HAS). Comment Améliorer le Parcours de Santé d'un Enfant Avec Troubles Spécifiques du Langage et des Apprentissages? Saint Denis: Guide Parcours de soins (2018). Available online at: https://www.has-sante.fr/upload/docs/application/pdf/2018-01/synthese_troubles_dys_v4.pdf (accessed on May 30, 2021).

[16] Hayes AM Dombrowski E Shefcyk A Bulat J. Learning Disabilities Screening and Evaluation Guide for Low- and Middle–Income Countries. Research Triangle Park: RTI Press Publication (2018). 10.3768/rtipress.2018.op.0052.1804 31449375

[17] Billard C Vol S Livet MO Motte J Vallée L Gillet P. The BREV neuropsychological test: Part I. Results from 500 normally developing children. Dev Med Child Neurol. (2002) 44: 391–98. 10.1017/S0012162201002262 12088307

[18] Billard C Motte J Farmer M Vol S Livet MO Vallée L. The BREV neuropsychological test: Part II. Results of validation in children with epilepsy. Dev Med Child Neurol. (2002) 44:398–404. 10.1017/s0012162201002274 12088308

[19] American Educational Research Association American Psychological Association National Council on Measurement in Education. Standards for Educational and Psychological Testing. Washington, DC: AERA publication (2014). p. 235.

[20] Nation K. Children's reading difficulties, language, and reflections on the simple view of reading. Austr J Learn Difficult. (2019) 24:47–73. 10.1080/19404158.2019.1609272

[21] Brendefur JL Johnson ES Keith WT Strother S Severson HH. Developing a multi–dimensional early elementary mathematics screener and diagnostic tool: the primary mathematics assessment. Early Childhood Educ J. (2018) 46:153–7. 10.1007/s10643-017-0854-x 29576730 PMC5859347

[22] Bishop DVM Snowling MJ Thompson PA Greenhalg T. Identifying language impairments in children. PLoS ONE. (2016) 11:158753. 10.1371/journal.pone.0158753 PMC4938414 27392128

[23] Billard C Mirassou A Touzin M. La Batterie Modulable de Tests Informatisée (BMT-i). Isbergues: OrthoÉdition (2019).

[24] INSEE Institut National de la statistique et des études économiques. Population Selon le Sexe et la Catégorie Socioprofessionnelle. Montrouge: Données annuelles de 2014 à 2019 (2020). Available online at: https://www.insee.fr/fr/statistiques/2381478

[25] Asselin AC Breton ML Elaboration d'un outil d'évaluation de la lecture proposé à 252 enfants. Recueil de Données Normatives. Paris: Mémoire d'orthophonie (1997).

[26] Boutard C Claire I Gretchanovsky L. Le vol du PC. Isbergues: OrthoÉdition. (2006).

[27] Baneath B Alberti C Boutard C Gatignol P. Chronosdictées. Isbergues: OrthoÉdition (2006).

[28] Chevrie–Muller C Maillart C Simon AM Fournier S. L2MA−2-Batterie langage oral, langageécrit, mémoire, attention – 2nde édition. Montreuil: ECPA par Pearson (2010).

[29] Weschler D. WISC–V. Échelle d'intelligence de Wechsler pour enfants et adolescents – 5e édition. Montreuil: ECPA par Pearson (2016).

[30] Nakagawa S Schielzeth H. Repeatability for Gaussian and non-Gaussian data: a practical guide for biologists. Biol Rev Camb Philos Soc. (2010) 85:935–56. 10.1111/j.1469-185X.2010.00141.x 20569253

[31] Stout W Froelich AG Gao F. Using resampling to produce an improved DIMTEST procedure. In: Boomsma A van Dujin MAJ Snijders TAB editors. Essays on Item Response Theory. New York, NY: Springer-Verlag (2001). p. 357–75.

[32] Jöreskog KG Sorböm D du Toit SHC du Toit M. LISREL 8: New Statistical Features (3rd Printing with revisions). Lincolnwood, IL: Scientific Software International, Inc. (2001).

[33] Cronbach LJ. Coefficient alpha and the internal structure of tests. Psychometrika. (1951) 16:297–334. 10.1007/BF02310555

[34] Peterson RA. A meta–analysis of Cronbach's coefficient alpha. J Consumer Res. (1994) 21:381–91. 10.1086/209405

[35] Schreiber JB Nora A Stage FK Barlow EA King J. Reporting structural equation modeling and confirmatory factor analysis results: a review. J Educ Res. (2006) 99:323–38. 10.3200/JOER.99.6.323-338

[36] Landis JR Koch GG. An application of hierarchical kappa–type statistics in the assessment of majority agreement among multiple observers. Biometrics. (1977) 33:363–74. 10.2307/2529786 884196

[37] SAS Institute Inc. JMP® 8 Statistics and Graphics Guide, Volumes 1 and 2. Cary, NC: SAS Institute Inc. (2008). Available online at: https://support.sas.com/documentation/onlinedoc/jmp/statguide_11147.pdf (accessed on May 30, 2021).

[38] R Core Team. R: A Language and Environment for Statistical Computing, R Foundation for Statistical Computing. Vienna (2020). Available online at: https://www.R-project.org/

[39] Enquête nationale périnatale (2017). Available online at: http://www.xn–epop-inserm-ebb.fr/wp-content/uploads/2017/10/ENP2016_rapport_complet.pdf (accessed May 30, 2021).

[40] Clanché F. Langues régionales, langues étrangères: de l'héritage à la pratique. Insee-première (2002). n°830. Available online at: http://www.epsilon.insee.fr:80/jspui/handle/1/459

[41] Stemler SE. A comparison of consensus, consistency, and measurement approaches to estimated interrater reliability. Pract Assess Res Evalu. (2004) 9:1–11. 10.7275/96jp-xz07

[42] Hulme C Snowling MJ. Reading disorders and dyslexia. Curr Opin Pediatr. (2016) 28:731–5 10.1097/MOP.0000000000000411 27496059 PMC5293161

[43] Nippold MA. Reading comprehension deficits in adolescents: addressing underlying language abilities. Lang Speech Hear Serv Schools. (2017) 48:125–31. 10.1044/2016_LSHSS-16-0048 28384784 PMC5544188

[44] Snyder L Caccamise D Wise B. The assessment of reading comprehension. Top Lang Disorders. (2005) 25:33–50. 10.1097/00011363-200501000-00005

[45] Ebert KD Ochoa–Lubinoff C Holmes MP. Screening school–age children for developmental language disorder in primary care. Int J Speech Lang Pathol. (2020) 22:152–62. 10.1080/17549507.2019.1632931 31262202 PMC6938570

[46] Barbiero C Montico M Lonciari I Monasta L Penge R Vio C. The lost children: the underdiagnosis of dyslexia in Italy. A cross–sectional national study. PLoS ONE. (2019) 14:e0210448. 10.1371/journal.pone.0210448 30673720 PMC6343900

[47] Fluss J Ziegler JC Warszawski J Ducot B Richard G Billard C. Poor reading in French elementary school: the interplay of cognitive, behavorial, and socioeconomic factors. J Dev Behav Pediatr. (2009) 30:206–16. 10.1097/DBP.0b013e3181a7ed6c 19412126

[48] Hellstranda H Korhonena J Räsänenc P Linnanmäkia K Auniob P. Reliability and validity evidence of the early numeracy test for identifying children at risk for mathematical learning difficulties. Int J Educ Res. (2020) 102:101580. 10.1016/j.ijer.2020.101580

[49] Wahlstrom D. Technology and computerized assessments: current state and future directions. In: Bush SS Demakis GJ Rohling ML editors. Apa Handbook of Forensic Neuropsychology. Washington, DC: APA PsycBooks (2017). p. 463–76. 10.1037/0000032-021

[50] Naz S Najam N. Neurological deficits and comorbidity in children with reading disorder. Psychiatry Clin Psychopharmacol. (2019) 29:674–81. 10.1080/24750573.2019.1589174

[51] Diamond A Ling DS. Executive functions. Ann Rev Psychol. (2013) 64:135–68. 10.1146/annurev-psych-113011-143750 23020641 PMC4084861

[52] Lin HY Chang WD Hsieh HC Yu WH Lee P. Relationship between intraindividual auditory and visual attention in children with ADHA. Res Dev Disabil. (2021) 108:103808. 10.1016/j.ridd.2020.103808 33242747

