# Supplementary material for: The Computerized Adaptable Test Battery (BMT-i) for Rapid Assessment of Children's Academic Skills and Cognitive Functions: A Validation Study
Source: Front Pediatr. 2021 Jul 8;9:656180. doi: 10.3389/fped.2021.656180 (PMC8295558; doi:10.3389/fped.2021.656180)
Supplement: Supplementary file 1 [file Data_Sheet_1.PDF]

frontiers

Supplementary Material

1 Supplementary Data

The BMT-*i* tests concern the five areas of academic aptitude and cognitive function. While the academic skill tests are specific to each age group, most of the cognitive function tests are identical for all classes within a given group, i.e., “youngest” (kindergarten through first grade), “intermediate” (second through fourth grades), or “oldest” (fifth through seventh grades) group. Scores are instantly and automatically converted into normed results summarized in a report (Supplementary Figure 1).

1.1 Written language (duration: 10 to 20 minutes, depending on age)

In the last year of kindergarten, the reading and dictation tests assess how well children know the letters of the alphabet. In first grade, acquisition of decoding skill was assessed by having children read letters, syllables, words, digraphs, trigraphs, pseudowords, and sentences of 3 to 6 words. In the third trimester, there was also a short text to read. From grades two to seven, one of two texts, of increasing length and difficulty, was used to evaluate reading speed, decoding errors, and comprehension (through open questions). The time of reading is automatically recorded, the decoding errors are reported in a specific document and the comprehension responses are directly recorded by the rater. Dictation tests evaluated phonetic, lexical, and grammatical aspects of orthography, and time to completion, using the same elements as the reading test, i.e., letters, syllables, words, pseudowords, sentences (first to fourth grades), or a text (fifth to seventh grades).

1.2 Mathematical cognition (duration: 10 to 20 minutes)

The content of the tests, selected according to the skills children in each age group are expected to acquire, targets each of the three major areas of mathematical cognition: numbers, arithmetic, and problem-solving. For each test, an accuracy score is assigned and the time to completion is recorded. Analog, number-word (accounting for the peculiarities of the French language, e.g., *quinze* and *quatre-vingt-dix*), and Indo-Arabic base-10 numerical representations are used. Starting in first grade, analog representations are explored together with base-10 numeral representations through the complementary C2C (“two-code comparison”) test. The child is shown 60 pairs of number representations in rapid succession and must determine whether both members of each pair are equivalent (Supplementary Figure 2). The child accuracy and time of responses are directly recorded. The relationship between number words and base-10 representations is explored through a dictation and a number reading test whose difficulty increases with the child’s age (*40; 8,070; 7.9; 506,037*). Starting in second grade, mental math fluency is evaluated by tallying the number of simple calculations performed in 1 min, for each type of arithmetic operation. There are three additional calculation tests: positioning of operands (for example, *7,502 − 408*), for each class; and problems expressed in words (for example, “5 is a third of ?”), or numerically (for example, "0.10 + 0.2 = ?”) for middle schoolers. For each class, children is asked to complete 5 to 8 math problems: the problems are visually represented from kindergarten through second grade; for older grades, a recording of the problem is played back by the test program. The score of each test was the number of good answers. For each class, a basic protocol is used to calculate a normed composite score assessing the child’s mathematical skill level.

1.3 Oral language (duration: 10 to 20 minutes)

Five tests analyzed oral language receptive and expressive skills (Supplementary Figure 3). The phonology test measures the child's capacity to analyze and repeat nonsense words. To evaluate lexical deployment, test takers are asked to name what is represented in each of 40 images. In order to evaluate lexical comprehension, the test program plays back recordings of 30 to 33 words and, for each, children must select the corresponding picture out of the six shown. Their responses are automatically recorded as touchscreen input. Syntactic expression is assessed through two tests. The first, administered to children from kindergarten to fourth grade, scores their ability to manipulate syntactic structures in order to complete sentences begun by the examiner. From second grade on, children must repeat 8 or 10 recorded sentences played back by the test program. This test assigns two scores: one for correct repetition of words and another for correct repetition of "syntactic morphemes." For example, the French sentence "Es-tu allé à ce spectacle auquel nous avions été invités ?" has 11 words and one syntactic morpheme (*auquel*). Comprehension of diverse syntactic notions is assessed by having children move virtual tokens on the touchscreen according to the directions given. The score is the number of good answers.

1.4 Nonverbal functions (duration: 15 to 20 minutes)

In this category, there are two fully computerized reasoning tests and four tests assessing drawing, handwriting, and visuospatial construction. The pattern completion test (Supplementary Figure 4), which explores fluid reasoning, consists of 20 to 24 matrices. Each matrix is made up of 4 to 6 boxes, all but one containing a picture. Children must identify the missing picture from a range of choices. Test takers' responses and the time taken to complete the task are automatically recorded when they select their choice on the touchscreen. The complementary classification test evaluates children's ability to categorize items according to nonverbal characteristics. There are 27 different emoticons, each defined by three variables: shape, color, and depicted emotion. Below these emoticons are three boxes: two contain emoticons that establish a conceptual pattern and the third is empty (Supplementary Figure 4). The test taker must complete the pattern by selecting the missing emoticon from among the 27 others.

To measure drawing quality and speed, children from kindergarten to fourth grade must copy 5 or 6 simple figures, and those in grades two to seven are asked to copy a complex figure. A handwriting score is assigned according to the quality of handwriting for the dictation, using six simple criteria. Visuospatial construction skills are evaluated on the basis of speed and accuracy for 15 block construction tasks.

1.5 Attention and executive functions (duration: 30 minutes)

Three fully computerized tests assess attentional and executive skills in children from grades two to seven. Sustained visual attention is assessed through a 15-minute go-no-go target detection test based on the paradigm of the Conners Continuous Performance Test 3rd Edition (Conners, 2014). Circles of different colors consecutively appear on the screen: every time a new circle is seen, the child must touch the blue bar that is displayed, unless that circle is black (Supplementary Figure 5). As black circles appear only 10% of the time (36 out of 360 circles), the test also evaluates the child's ability to check the acquired reflex of touching the blue bar. An algorithm calculates various scores on the basis

of the touchscreen input. The percentage of negative errors or *omissions* (i.e., instances when the child failed to touch the bar when a colored circle was shown) indicates ability to sustain attention. The percentage of positive errors or *commissions* (i.e., instances when the child erroneously touched the bar when a black circle was shown) is an indicator of impulsivity. The median reaction time is a measure of speed of response, and the standard deviation of the reaction times is an indicator of their variation.

The two-part, 8-minute controlled auditory attention test is aimed at evaluating selective attention as well as inhibition and flexibility, through “control” (part A) and “conflict” (part B) tasks. Four shapes are displayed on the screen (Supplementary Figure 5), and the test taker hears a sequence of words, including the names of the on-screen shapes. For the “control” task (part A), the child must touch the circle as quickly as possible every time the word “circle” is heard, and do nothing when another word is heard. For the “conflict” task (part B), the child must remember several instructions, touching the circle when the word “square” is heard, and vice versa; the triangle when “triangle” is heard; and no shape in other cases. The inverted instructions for circles and squares demand that the child check the logical reflex, while the instruction for triangles, on the contrary, demands such a reflex. An algorithm converts the touchscreen input into an accuracy score for the control task and three scores for the conflict task: a “flexibility” score evaluating execution of the square/circle instruction, a “triangle” score for the triangle instruction, and a reflex inhibition error score.

The digit span (French *empans*) test has children repeat successive series of 3 to 7 digits in forward and backward orders. The program announces the digits at a constant speed of one per second. This test assesses short-term memory and the ability to manipulate memorized information.

1.6 Supplementary Figures

Figure 1 Example of a test report (1st grade normal child-3rd trimester)
Scores are instantly and automatically converted into normed results summarized in a report. CA: correct answers; sec: seconds. WCR/mn = number of words correctly read per minute.

Figure 2 Two-codes comparison test (C2C): base-10 numeral analog representations are compared with analog representations
Consigne : « Regarde, est-ce que c’est la même quantité ? Appuie sur oui ou non. » : *Instruction : « Look, is it the same quantity ? Press yes or no. »*

Figure 3 Example of pictures (language oral skills): lexical comprehension (*uniform)* and syntactic comprehension (« *Among the circle, touch the blues* »).

Figure 4 Reasoning tests: screenshot of an item of pattern completion test and screenshot of classification test

Figure 5 Screenshot of visual attention test and auditory attention test
