# Supplementary figures and images for: The Computerized Adaptable Test Battery (BMT-i) for Rapid Assessment of Children's Academic Skills and Cognitive Functions: A Validation Study
Source: Front Pediatr. 2021 Jul 8;9:656180. doi: 10.3389/fped.2021.656180 (PMC8295558; doi:10.3389/fped.2021.656180)

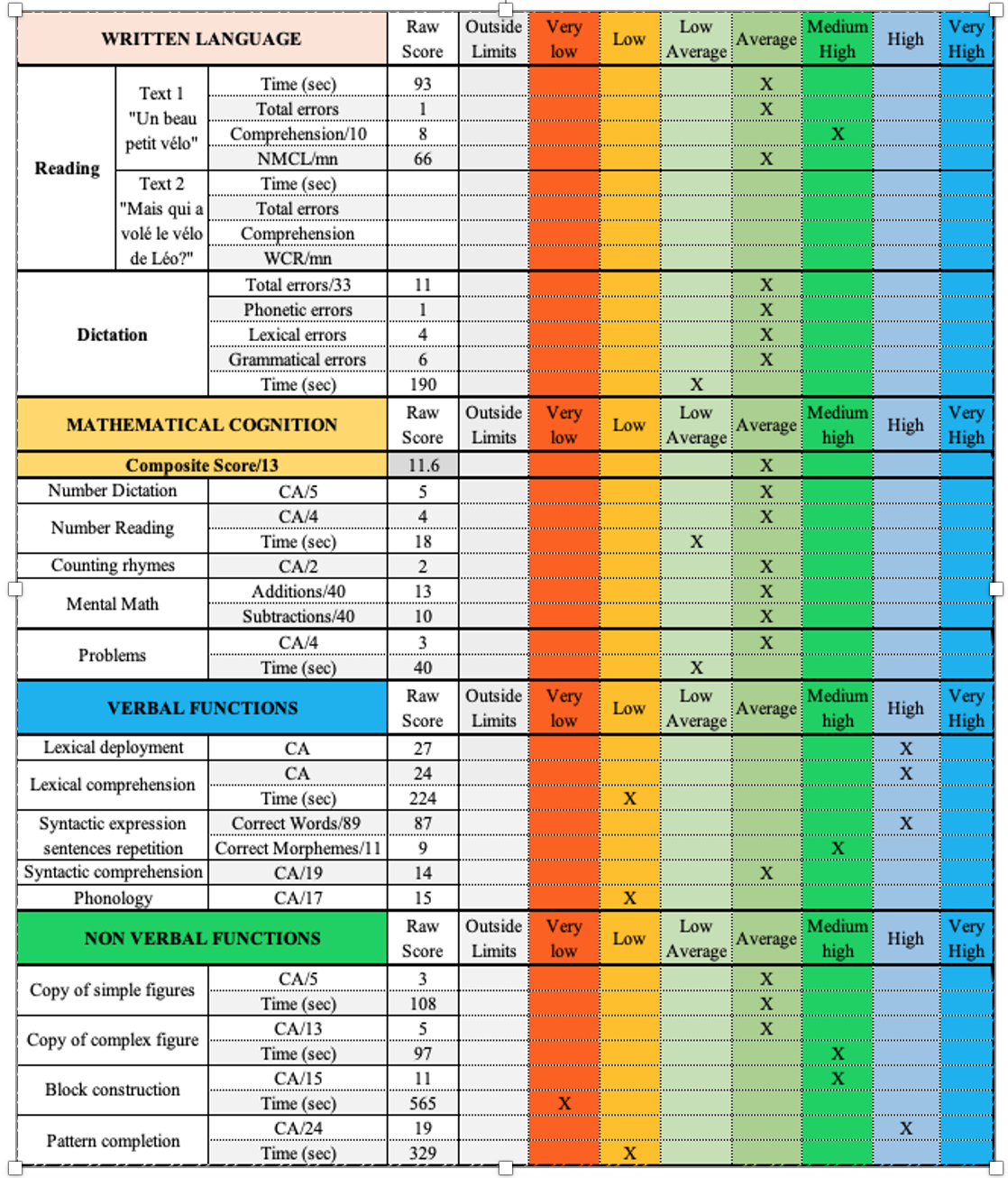

Supplement: Supplementary file 2 [file Image_1.JPEG]

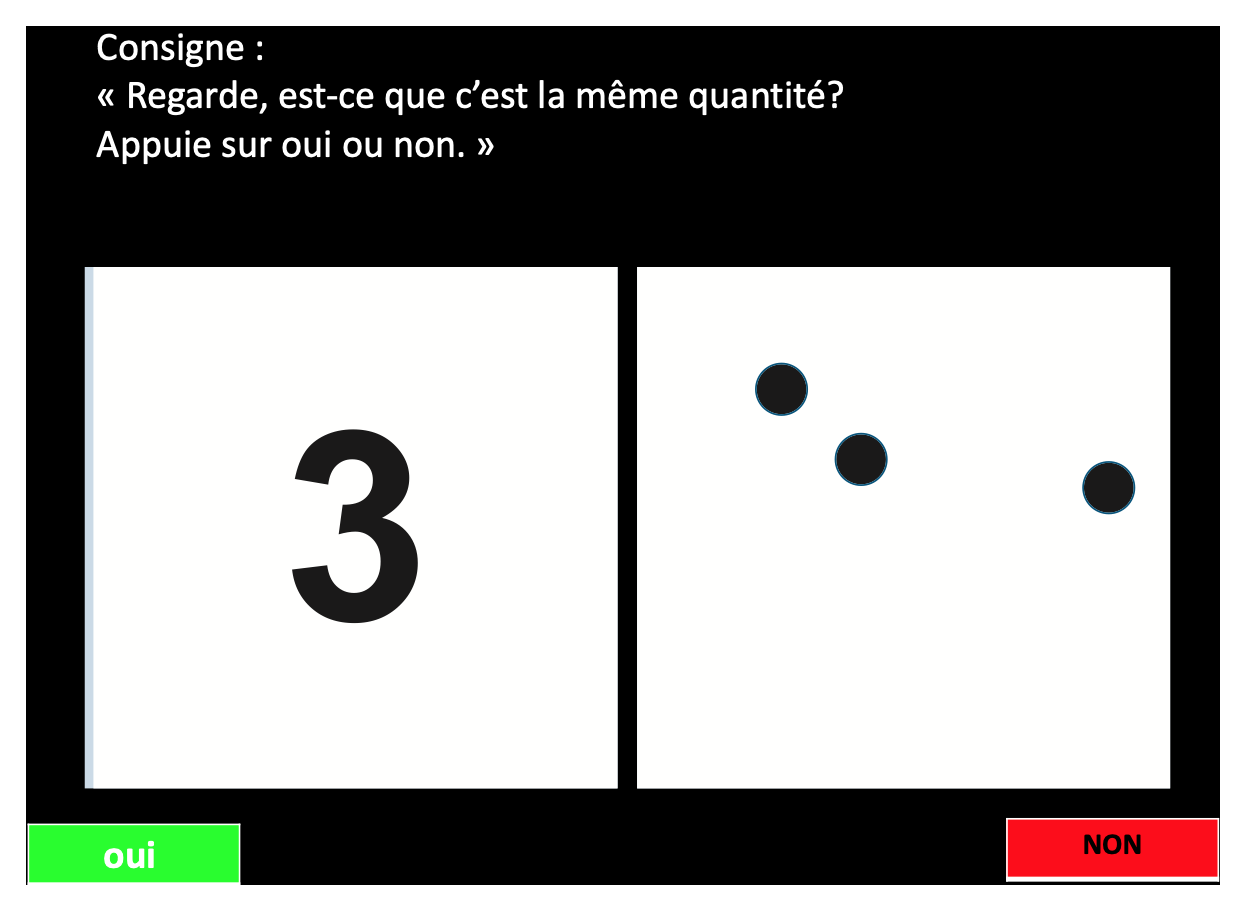

Supplement: Supplementary file 3 [file Image_2.JPEG]

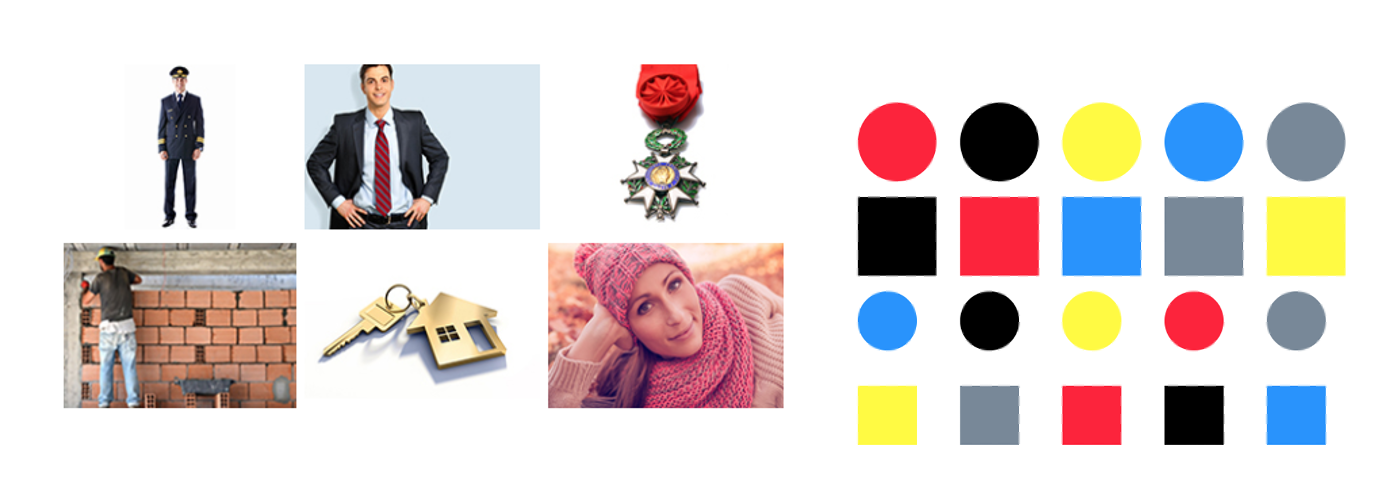

Supplement: Supplementary file 4 [file Image_3.JPEG]

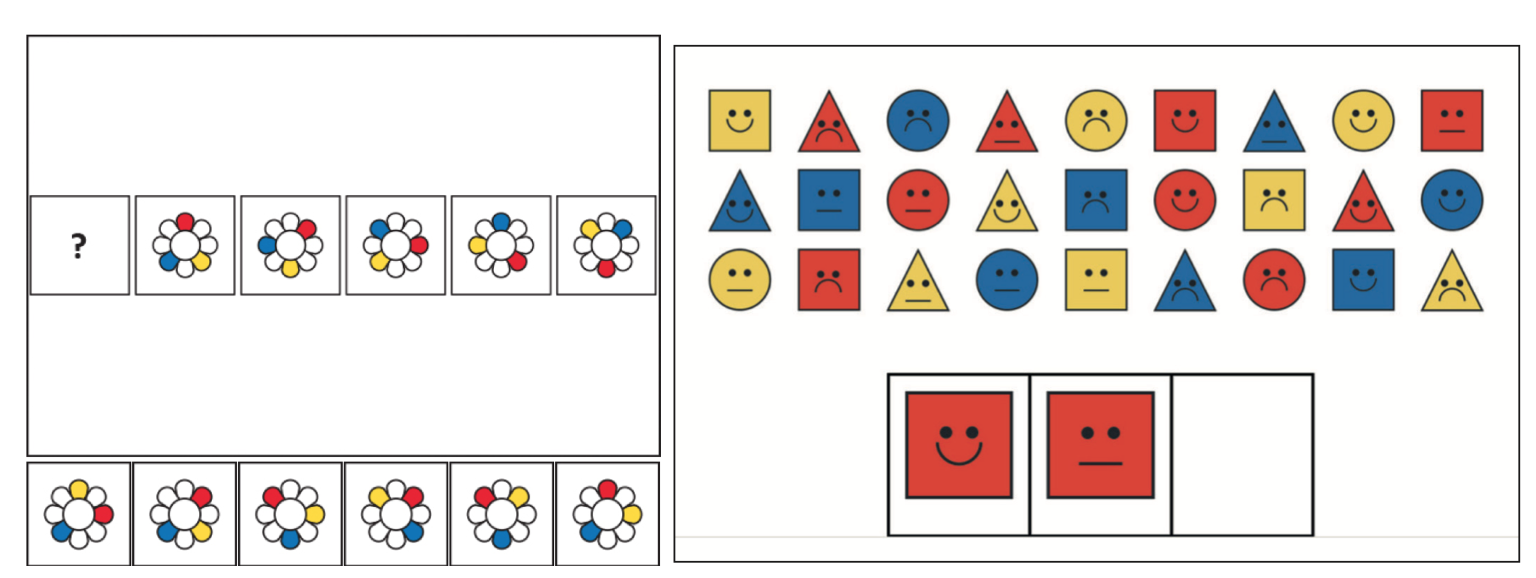

Supplement: Supplementary file 5 [file Image_4.PNG]

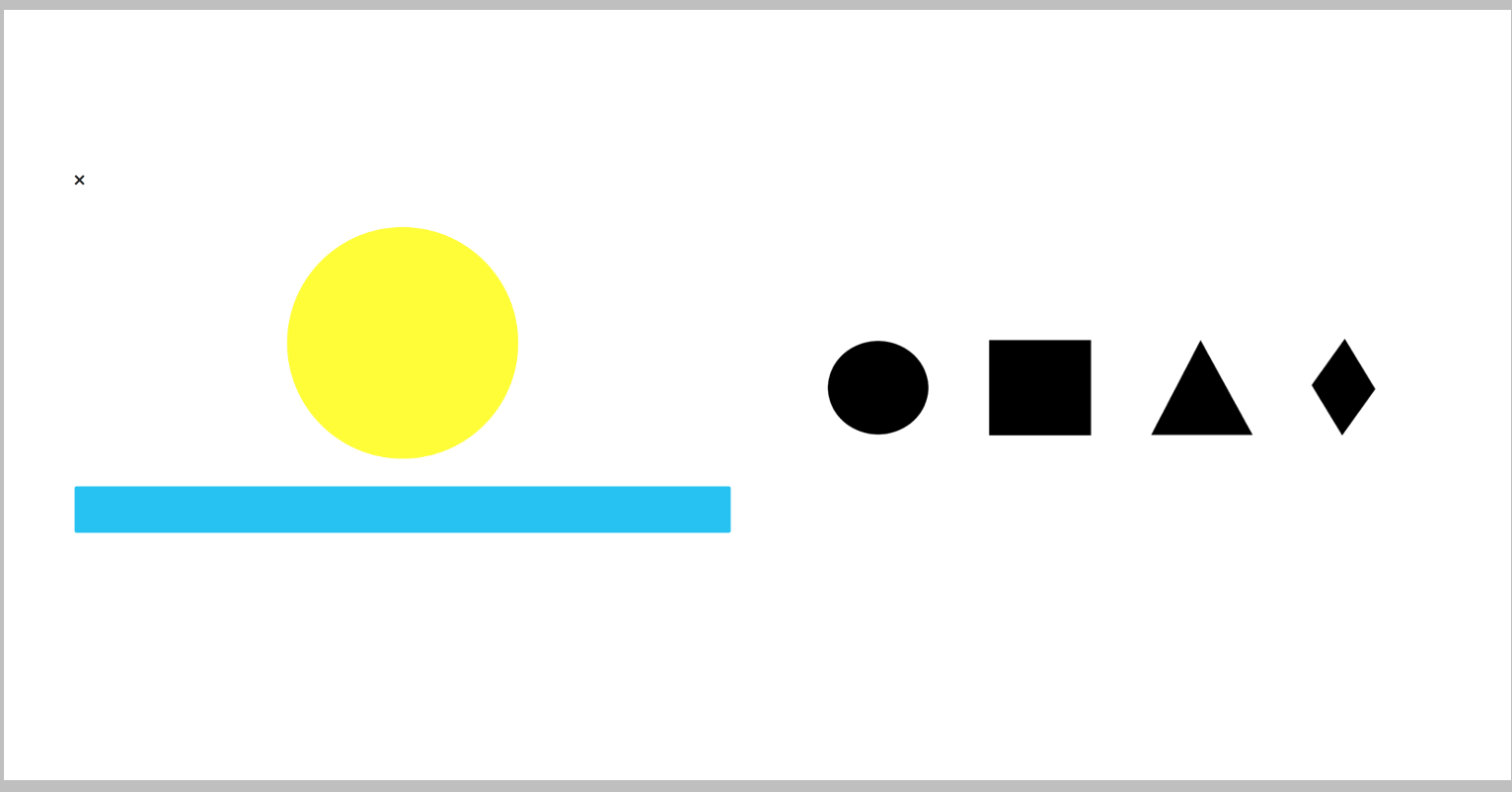

Supplement: Supplementary file 6 [file Image_5.JPEG]
